# Harmonisation of quality control tests for academic production of CAR-T cells: a position paper from the WP-bioproduction of the UNITC consortium

**DOI:** 10.1038/s41409-025-02637-8

**Published:** 2025-05-29

**Authors:** Chrystel Marton, Béatrice Clémenceau, Guillaume Dachy, Clémence Demerle, Sophie Derenne, Christophe Ferrand, Camille Giverne, Jean-Baptiste Latouche, Ludovic Lemée, Jérémie Martinet, Halvard Bonig, Danièle Bensoussan, Christian Chabannon, Ulrike Köhl, Marina Deschamps, John De Vos, Jean-Sébastien Diana, Aurore Dougé, Edouard Forcade, Jeanne Galaine, Stéphanie Thiant, Anne Galy, Jérôme Larghero, Loïc Reppel, Sébastien Viel, Olivier Boyer, Ibrahim Yakoub-Agha

**Affiliations:** 1 CHU Lille, Univ. Lille, Inserm, U1286 - INFINITE - Institute for Translational Research in Inflammation, F-59000 Lille, France; 2 Unité de Thérapie Cellulaire et Génique (UTCG), CHU de Nantes, Nantes, France; 3 Hematology department, Cliniques Universitaires Saint-Luc, Brussels, Belgium; 4 Centre de Thérapie Cellulaire, Institut Paoli-Calmettes (IPC) & module Biothérapies du Centre d’Investigations Cliniques de Marseille, Inserm CBT-1409 / Aix-Marseille Université / AP-HM / IPC, Marseille, France; 5 Etablissement Français du Sang, La Plaine Saint Denis, Saint-Denis, France; 6 Université de Franche-Comté, EFS, INSERM, UMR RIGHT, F-25000 Besançon, France; 7 CHU de Rouen, Laboratoire d’Immunologie et de Biothérapies, Université de Rouen Normandie, Inserm, UMR 1234, F-76000 Rouen, France; 8 Univ Rouen Normandie, Inserm, UMR 1234, CHU de Rouen, Department of Immunology and Biotherapy, F-76000 Rouen, France; 9 CHU de Rouen, Department of Virology, F-76000 Rouen, France; 10 Goethe University, Faculty of Medicine, Institute for Transfusion Medicine and Immunohematology, Frankfurt a.M, Germany; 11 CHRU de Nancy, Unité de Thérapie cellulaire et Banque de Tissus (UTCT). Université de Lorraine, UMR 7365 IMoPA, Vandoeuvre-les-Nancy, France; 12 Fraunhofer Institute for Cell Therapy and Immunology as well as Institute for Clinical Immunology, University of Leipzig, Leipzig, Germany; 13 Department of Cell and Tissue Engineering, University Montpellier, CHU Montpellier, F-34000 Montpellier, France; 14 APHP, Hôpital Universitaire Necker Enfants Malades, Centre d’Investigation Clinique en Biothérapie, Laboratoire de Thérapie Cellulaire et Génique, Paris, France; 15 CHU de Clermont-Ferrand, Service Oncologie médicale, EA 7453 Chelter Université Clermont Auvergne, 63000 Clermont-Ferrand, France; 16 CHU de Bordeaux, Service Hématologie Clinique et Thérapie Cellulaire, UMR 5164, F-33000 Bordeaux, France; 17 Division of hematology, Oncology and transplantation, department of medicine, Maisonneuve-Rosemont hospital, Montréal, QC Canada; 18 ART-TG, Inserm, US35, 91100 Corbeil-Essonnes, France; 19 AP-HP, Université Paris Cité, Hôpital Saint-Louis, Centre MEARY de Thérapie Cellulaire et Génique, Unité de Thérapie Cellulaire, Centre d’Investigation Clinique en Biothérapies, Inserm, Paris, France; 20 Plateforme de biothérapies - Médicaments de Thérapie Innovante, Hôpital Edouard Herriot, Hospices Civils de Lyon, 69003 Lyon, France

**Keywords:** Gene therapy, Cancer therapy

## Abstract

This position paper from the Bioproduction Working Group of the UNITC Consortium seeks to harmonize quality control (QC) procedures for academic production of autologous CAR-T cells. The primary objective is to standardize QC testing for batch release in academic cell therapy units. Academic CAR-T manufacturing under the hospital exemption pathway enables faster, more cost-effective production and the use of fresh cells, eliminating the need for cryopreservation. Standardized QC processes are critical to ensure consistent product quality and safety. This paper focuses on key QC measures, including mycoplasma detection using validated commercial kits or in-house methods with on-site validation, endotoxin testing via Limulus Amebocyte Lysate (LAL) or Recombinant Factor C (rFC) assays with validated protocols to prevent matrix interference, vector copy number (VCN) quantification through validated qPCR or ddPCR techniques, and potency assessment through IFN-γ ELISA following antigenic stimulation. Emphasizing method validation and standardized testing, this work underscores the importance of robust QC strategies to ensure the safety and efficacy of CAR-T cell therapies, with ongoing efforts dedicated to optimizing these processes. This workshop focuses on addressing the harmonization of some quality control (QC) measures required for the validation of academic CAR-T cell production :mycoplasma detection; endotoxin testing; vector copy number (VCN) quantification; potency testing and the use of surrogate markers, if applicable. Sterility testing and characterization/identity/purity assessments are not covered in this work.

## Introduction and state of the art

Cell and gene therapy medicinal products are part of innovative therapies that involve the use of living cells, modified genes, or viral vectors to treat serious or chronic diseases. The quality and safety of these therapies are crucial to ensuring their therapeutic effectiveness and safety.

CAR-T cells represent a significant advancement in the field of cell and gene therapy. Although they originated from academic research, CAR-T cells have gained considerable interest from the pharmaceutical industry, spreading primarily across North America, China, and Western Europe. However, access to these therapies remains limited due to high production costs, complex logistics, and, in some cases, the saturation of centralized production facilities [1, 2].

European Regulation EC No. 1394/2007 of November 13, 2007, established the hospital exemption clause for Advanced Therapy Medicinal Products (ATMPs). This clause defines the conditions under which ATMPs can be manufactured by a healthcare institution for a specific patient under the responsibility of a medical practitioner and used within the same EU member state after approval by the regulatory authority, while adhering to quality standards equivalent to those of ATMP manufacturing. With the approval of the French National Agency for Medicines and Health Products Safety (ANSM), French academic cell therapy units can produce CAR-T cells [3].

Academic production of autologous CAR-T cells offers several potential advantages, such as faster and more cost-effective production and a more efficient pharmaceutical supply chain. This approach leverages existing, functional workflows within academic institutions (also known as “Cell Processing Facilities” under the international FACT-JACIE standards for hematopoietic cell therapies) that can coordinate all stakeholders involved in preparing a patient-specific cell product. Additionally, local academic production enables the administration of fresh cell products, avoiding the cryopreservation/thawing step, which poses a risk of quantitative and qualitative loss of CAR-T cells. In a Phase 1 dose escalation and expansion trial using fresh bispecific anti-CD20/anti-CD19 CAR T cells for relapsed B-cell malignancies, investigators found that on-site manufacturing and infusion of non-cryopreserved CAR T cells were both feasible and therapeutically safe, demonstrating low toxicity and high efficacy [4]. In addition, we could show homogenous results in the manufacturing of CD20 CAR T cells freshly used in a clinical phase I melanoma trial produced at two different manufacturing sides [5]. Although improvements in cryopreservation techniques may reduce the efficacy gap between fresh and cryopreserved CAR-T products, these results underscore the potential advantage of on-site cell manufacturing. However, scaling this practice across numerous medical centers while ensuring consistent product quality remains a major challenge.

In recent years, the development of semi-automated, closed-system production devices has emerged as a promising solution to facilitate the deployment of CAR-T cell manufacturing in academic units [6, 7]. Furthermore, academic production can address a broader range of indications, particularly rare diseases not covered by the marketing authorizations (MAs) of commercial CAR-T products, thus offering more patients access to these therapeutic advances. While the simplicity of CAR-T cell production using automated bioreactors with closed systems enables broader manufacturing access, quality control remains a significant hurdle due to its complexity, especially concerning potency assessment. Ai-driven automated modular production lines as well as automated QC with inline sensors are of paramount importance [8]. Additionally, as CAR-T cells are classified as medicinal products, their point-of-care manufacturing must be integrated into a robust pharmaceutical quality management system [9].

To support the development and accessibility of CAR-T cells in France, the WP3-Bioproduction group of the UNITC Consortium (National Consortium for Research on Cell Therapies), bringing together experts in academic production, was established at the end of 2023.

Our group has initiated a series of workshops aimed at systematically and rigorously reviewing all critical steps of the typical manufacturing process for autologous CAR-T cells, with the goal of harmonizing production and quality control practices and supporting standardization among academic manufacturing facilities. The objective of this work is to harmonize the use of validated quality control tests necessary for the release of autologous CAR-T cell batches across academic cell therapy units. These tests include mycoplasma detection, endotoxin testing, vector copy number (VCN) quantification, and potency testing.

## Methodology

This workshop was conducted following the methodology developed by the Société Francophone de Greffe de Moelle et de Thérapie Cellulaire (SFGM-TC) and recommended by the European Society for Blood and Marrow Transplantation (EBMT) [10, 11].

The work was based on regulatory frameworks such as Good Manufacturing Practices (GMP), the European Pharmacopoeia, guidelines from the European Medicines Agency (EMA), and directives from the International Council for Harmonisation (ICH) (Eudralex Volume 4, EMA Guidelines for ATMPs, ANSM GMP Guide) [12] as well as guidelines from the U.S. Food and Drug Administration (FDA). This approach also included a literature review [13–22] and an analysis of current practices through a national survey and expert discussions.

Following an initial workshop on identifying the conditions and minimum requirements for the release of autologous fresh CAR-T cell products under hospital exemption [23], the WP3-Bioproduction group of the UNITC Consortium launched a second workshop focused on developing recommendations to harmonize key quality control tests for CAR-T cell batch release. The next phase involved a series of teleconferences to refine and advance the project. Within each workshop subgroup, participants conducted comprehensive literature reviews to establish a solid foundation for further discussions. The process culminated in a two-day in-person meeting in Lille, France, in October 2024, where this document was drafted.

## Mycoplasma detection

In Europe, the absence of mycoplasmas in cell and gene therapy products is a regulatory requirement aimed at ensuring the safety of biological products intended for human use, as recommended by the European Pharmacopoeia. Other international pharmacopoeia standards, such as the United States Pharmacopeia (USP) and the Code of Federal Regulations, as well as the Japanese Pharmacopoeia (JP), may also apply depending on the geographical distribution of the products. The reference test for mycoplasma detection is defined in Chapter 2.6.7 of the European Pharmacopoeia, USP < 63 > , and JP XVIII. This test requires culturing mycoplasmas using a combination of three methods: broth culture, solid permissive medium culture, and fluorescent antibody detection of non-cultivable organisms grown on a cell monolayer.

The long turnaround time (28 days) and the large product volume (~15 mL) required for the reference test are not suitable with biological products that have a short shelf life (48 to 72 h). Alternative tests are accepted provided they are validated against the reference methods. Nucleic acid amplification techniques are considered an acceptable alternative for mycoplasma detection in cell and gene therapy products. Several commercial kits are available for mycoplasma detection, and internally developed tests may also be used if validated in accordance with current regulatory requirements (Table 1) (ICH Official web site: ICH).

**Table 1:** Comparison between commercial kits and in-house developed methods for mycoplasma detection.

Parameter	Biofire Mycoplasma (Biomérieux)	Commercial PCR Kits (e.g., Lifetech MycoSeq (Thermofisher), MycoTOOL (Roche), VenorGEMq oneStep (Minervabiolabs), MicroSART® (Sartorius))	In-house developed qPCR or ddPCR method
**Commercial Analytical Kit**	Yes	Yes	No
**Compliance with EP 2.6.7**	Yes	Yes	According to the validation performed
**Sensitivity**	<10 CFU/mL (as stated by the supplier)	<10 CFU/mL (as stated by the supplier)	According to the validation performed
**Method Validation**	* Validation performed by the supplier across the entire test, including the extraction kit. * In-house validation required: detection limits and no interference with each matrix used.	* Validation performed by the supplier across the entire test, including the extraction kit and thermocycler. * In-house validation required: detection limits and no interference with each matrix used.	* Full validation to be performed by the laboratory, in accordance with ICH Q2 guidelines. * This validation will include no interference with each matrix used.
**Number of Strains Detected**	>120 strains	Between 70 and 140 strains depending on the kit	At least the strains recommended by the EP. According to method validation.
**Specific Equipment Required**	Yes However, if the thermocycler used is not the one used for validation by the supplier, more extensive in-house validation will be required	No	No
**Implementation Difficulties**	+	+/++	+++ (related to method validation)
**Time from sample to result**	1-hour assay	Under 5 h	Under 5 h
**Cost**	+++++	++	+

Main criteria for selecting a nucleic acid amplification test are the following.

1. Compatibility of DNA extraction with the amplification method: the DNA extraction step is included in the overall validation of a test. It is important to select the extraction method/kit used during the test’s validation to ensure the supplier’s validation remains effective.
2. Validation matrix on cells and/or supernatant: the test should be validated for both cell suspensions and culture supernatants to reflect real production conditions.
3. Detection of mycoplasma strains recommended by the Pharmacopoeia: the proposed list of strains represents an optimal selection but can be adapted based on the risk analysis related to the production process.
4. Sensitivity/limit of detection for each strain: can the test detect at least 10 CFU/mL for each targeted strain?
5. High specificity to prevent false positives: certain kits, due to their targeted sequences, may cross-react with bacterial DNA, leading to false-positive results. However, in such cases, the sterility test would also be positive, resulting in the batch failing certification.
6. Sample handling and compatibility with the production system: the test must be suitable for the sample types and volumes used in the specific production workflow.
7. Ease of use and speed of results: the method should be user-friendly and provide rapid results to meet production timelines.
8. Overall cost per test: the total cost should account for reagents, labor, and equipment use, ensuring it aligns with budget constraints.
9. Access to required equipment for the selected test: the necessary equipment (e.g., thermocyclers, extraction systems) must be available and compatible with the chosen test method.

In the case of commercial kits, a validation report is generally provided by the supplier. However, in-house method validation is essential, particularly to reverify detection limits and validate the matrices used.

### Group recommendations

Given the use of GMP-certified reagents for CAR-T cell production process, as well as a controlled, semi-closed environment, the risk of mycoplasma contamination is considered reduced. Based on extensive clinical experience with hematopoietic cell transplants and the risk of patient contamination by mycoplasma, it seems reasonable to use commercial kits that have already been validated according to the European Pharmacopoeia, in the absence of a microbiology service with the appropriate expertise and resources. However, it is essential for each user to perform the necessary validation tests at the local level. Indeed, kits are validated using specific equipment, and it is important to ensure that the expected performance is achieved under real-use conditions, particularly regarding detection limits and matrix validation. The following steps are recommended for this limited in-house validation when using a commercial kit.

- Obtain a representative strain panel recommended by the Pharmacopoeia. Commercial reference strain standards with predefined concentrations (10 CFU/mL) are available.
- Use CAR-T cell suspension (cells and supernatant) as a matrix.
- For the use of CAR-T cell production, the group recommends completing the supplier validation of the commercial kit to control sensitivity on the cell suspension, as the aim is to demonstrate the absence of any mycoplasma or bacterial contamination.
- In cases where production is carried out across multiple sites using the same matrix and commercial kit, the validation data can be partially shared: a minimum verification of the thresholds, using the site’s equipment and within the laboratory environment, remains necessary.

## Endotoxin testing

The endotoxin assay is essential for detecting the presence of bacterial pyrogenic contaminants that can cause severe reactions in patients, including fever, septic shock, and, in extreme cases, death. Detecting and quantifying endotoxins in cell and gene therapy products that have undergone a culture step is crucial for ensuring patient safety, as even low concentrations can trigger adverse reactions, particularly in immunocompromised patients, such as those treated with CAR-T cells.

Regulatory agencies, such as the FDA and EMA, impose strict standards for endotoxin detection in cell and gene therapy products. Endotoxin detection limits are set based on the route of administration, administered volume, and patient condition. Generally, for injectable products, the limit is 5 EU/kg/hour (endotoxin units per kilogram of body weight per hour). It is important to note that one International Unit (IU) of endotoxin is equivalent to one Endotoxin Unit (EU).

For cell and gene therapy products, specific guidelines such as ICH Q5A and the European Pharmacopoeia (chapter 2.6.14) outline the expectations for endotoxin testing. Product developers must demonstrate the validation of the methods used, including proof of the absence of matrix interference, and adapt their methods to the specific characteristics of their products.

### Endotoxin assay methods

#### Limulus amebocyte lysate (LAL) [24, 25]

The most commonly used test for detecting endotoxins is the Limulus Amebocyte Lysate (LAL) assay, which relies on the coagulation of horseshoe crab amebocyte lysates in the presence of endotoxins. There are three main variants of the LAL test.

- Gel-Clot method: gel formation in the presence of endotoxins indicates a positive result. This method is simple but less sensitive and may lack precision in complex matrices like cell-based products.
- Chromogenic method: based on the release of a dye in the presence of endotoxins, this method is more sensitive than the gel-clot method and allows for more precise quantification.
- Turbidimetric method: measures turbidity caused by lysate aggregation in the presence of endotoxins. It offers intermediate sensitivity and is faster, making it useful for high-throughput testing.

#### Test recombinant factor C (rFC) [26]

An alternative to the LAL test based on recombinant proteins (recombinant Factor C) has been developed. This test avoids the use of horseshoe crabs, making it more ethical and sustainable. The rFC assay offers performance comparable to the LAL test and is recognized by some regulatory agencies as a valid method for endotoxin detection.

Table 2 presents a comparison of the two endotoxin testing methods.

**Table 2:** Comparison of the two endotoxin testing methods.

Method	Advantages	Limitations
**LAL**	- Widely used method. - Three variants available (Gel-clot, Chromogenic, Turbidimetric), providing flexibility based on needs. - The chromogenic method is more sensitive than the gel-clot method and allows for more precise quantification. - The turbidimetric method is faster, making it useful for high-throughput testing.	- The gel-clot method is less sensitive and may lack precision in complex matrices such as cell-based products. - Cellular components or excipients may interfere with the test by masking the presence of endotoxins or disrupting the lysate-endotoxin reaction.
**rFC**	- Avoids the use of horseshoe crabs, making it more ethical and sustainable. - Offers performance comparable to the LAL test. - Recognized by some regulatory agencies as a valid method for endotoxin detection.	- No specific disadvantages have been mentioned in sources, but as an alternative to the LAL test, it may face similar challenges regarding matrix interference in complex products.

Abbreviations: *LAL* Limulus Amebocyte Lysate; *rFC* Recombinant Factor C.

### Challenges of endotoxin assays in CAR-T cell batches

Cell and gene therapy products present unique challenges for endotoxin testing, primarily due to their complexity and the nature of the biological matrices used.

Matrix interferences must be considered, as CAR-T cell products contain proteins, living cells, and other biomolecules that can interfere with endotoxin tests by inhibiting or distorting the results of LAL-based methods. For example, cellular components or excipients may mask the presence of endotoxins or disrupt the lysate-endotoxin reaction.

Moreover, CAR-T cell products, particularly autologous ones, are often produced in small quantities. This makes quality testing challenging, as each test requires a certain volume of the final product, potentially reducing the amount available for the patient.

Finally, the sensitivity of endotoxin testing methods must be adapted to the specific characteristics of CAR-T cell products. Detection levels must be sufficiently low to ensure patient safety, while the method must also be specific enough to avoid false positives in the presence of other biological components.

### Group recommendations

#### Method validation

Validating endotoxin assay methods is essential to ensure the test performs reliably with complex matrices. This validation involves assessing potential interferences, detection limits, and the reproducibility of the test.

#### Verification of the absence of endotoxin

In manufacturing, preventing endotoxin contamination is critical. Using pharmaceutical-grade materials, water for injection, and equipment sterilized through validated methods (such as dry heat or filtration) is crucial to minimize contamination risks.

#### Use of recombinant products

The rFC assay can help overcome some limitations of traditional LAL testing, particularly regarding sensitivity in complex matrices. Additionally, reducing biological variability from horseshoe crab lysates allows for greater standardization.

#### Result reporting

Endotoxin assay results are typically reported in EU/mL of the tested product. This result should be converted to EU/kg based on the patient’s weight and the intended dosage.

## Quantification of vector copy number (VCN)

The modification of T lymphocytes is typically achieved using a viral vector (gammaretroviral or lentiviral) that enables the integration of the CAR receptor gene into the cells’ genome. Quantifying the VCN, the number of vector copies integrated into the cell genome, is a critical step for several reasons.

- Therapeutic Efficacy: an insufficient number of vector copies may result in low or absent CAR receptor expression, compromising product efficacy [27–30].
- Safety: an excessive number of vector copies can lead to CAR overexpression, increasing toxicity risks. Additionally, a high copy number raises the risk of insertional mutagenesis, where the vector integrates into genomic regions that could activate proto-oncogenes or disable tumor suppressor genes [28–31].

Regulatory agencies enforce strict standards for VCN quantification to ensure patient safety. For example, the FDA requires that quantification methods be validated and provide accurate data on the number of integrated copies per cell. Manufacturers must demonstrate that their method can detect potentially hazardous VCN levels that could increase the risk of insertional mutagenesis or CAR overexpression-related toxicity. Regulatory limits on VCN may vary depending on the therapy and vector used. Typically, agencies mandate close VCN monitoring during early clinical trial phases and require long-term studies to track potential effects of genetic modification.

Of note, academic production of CAR-T cells offers a significant advantage in VCN quantification, as the same method can be applied both to the production process and patient monitoring.

### VCN quantification methods

Several techniques are used to quantify VCN in CAR-T cells, with the most common being quantitative PCR (qPCR) and droplet digital PCR (ddPCR).

- qPCR: this standard method quantifies VCN by amplifying a specific vector sequence (either from the construct itself or a common backbone shared by many vectors). Real-time quantification is achieved by measuring fluorescence emitted by fluorochrome-labeled probes during amplification.
- ddPCR: a more recent and precise method for VCN quantification, ddPCR partitions a DNA sample into thousands of droplets, each containing one or more DNA molecules. Amplification occurs individually within each droplet, enabling absolute copy number quantification without the need for standard curves.
- Alternative methods: less common techniques include deep sequencing for VCN quantification and Fluorescence In Situ Hybridization (FISH). These methods are generally reserved for research analyses or long-term safety studies and may be used in cases of unexpected complications following CAR-T cell therapy.

Table 3 presents advantages and limitations of qPCR and ddPCR for VCN quantification.

**Table 3:** Advantages and limitations of VCN quantification techniques.

Test	Advantages	Limitations
**qPCR**	Well-established and validated method. Relatively fast and accessible.	qPCR is an indirect method that requires the use of standards for quantification. It can be subject to variations depending on amplification efficiency and biases introduced during analysis.
**ddPCR**	Absolute quantification of VCN, eliminating the need for a standard curve. High precision, sensitivity, and reproducibility. Less prone to variations than qPCR, allowing detection of copy number variations even in heterogeneous samples.	Expensive method requiring specialized equipment. May be slower than qPCR in some laboratories.

*qPCR* PCR Quantitative, *ddPCR* Droplet Digital PCR.

### Challenges in VCN quantification in CAR-T Cell batches

The VCN quantification in CAR-T products presents several technical and biological challenges.

- Sample heterogeneity: CAR-T products often consist of heterogeneous cell populations, with modified cells containing varying amounts of vector copies. This variability can complicate quantification and lead to significant result fluctuations. It is therefore crucial to use sensitive methods capable of accurately detecting and quantifying cell populations with variable VCNs. Additionally, VCN quantification varies depending on the vector used in the production process.
- Limited biological material: since CAR-T products are manufactured from the patient’s own cells (in the case of autologous products), the available material for testing is often limited. Laboratories must adapt their methods to ensure that VCN quantification can be performed without consuming an excessive portion of the final product.
- Standardization and validation of methods: one of the major challenges lies in standardizing the methods used for VCN quantification. Each laboratory or production center may use different reagents, equipment, and protocols, potentially leading to variations in results. Therefore, it is essential to validate each method to ensure it meets the necessary requirements for accuracy, sensitivity, and reproducibility.

### Group recommendations

#### Rigorous method validation

Validating quantification methods, whether through qPCR or ddPCR, is essential to ensure reliable results. This includes assessing the sensitivity, specificity, linearity, and reproducibility of the tests. A thorough validation process helps minimize batch-to-batch variations and ensures result consistency.

#### Protocol harmonization

To reduce variability between laboratories, harmonizing VCN quantification protocols is encouraged. This may involve adopting standardized methods such as ddPCR and using shared reference materials among production centers to ensure result comparability. However, harmonization can be challenging, as reagents must be adapted to the vector used for each genetic modification (which is easier with backbone-specific probes).

#### Multimethod approach

In some cases, using multiple complementary methods for VCN quantification may be beneficial. For example, an initial qPCR analysis can be followed by a more precise validation using ddPCR for high-risk products. Additionally, further safety tests, such as deep sequencing, may be considered to assess potential risks of insertion into critical genomic regions.

## Potency test

The potency test ensures that a product meets predefined biological functional characteristics before batch release for clinical use. In the case of CAR-T cells, this test assesses their ability to recognize the CAR’s specific target antigen by measuring one of three induced lymphocyte functional activities: cytotoxic activity, cytokine production, or proliferation. A quantitative evaluation of one of these functional activities helps validate the manufacturing process and ensures the quality of the final CAR-T cell preparation. This test can also be used to validate a freezing/thawing step and to conduct stability studies. In the best-case scenario, although not its primary purpose, the potency assay could also serve as a predictive biomarker of clinical efficacy in treated patients.

Several biological factors associated with favorable clinical outcomes in CAR-T cell therapy have been identified. For instance, the dose of reinfused CAR-T cells has been suggested to influence clinical outcomes [32, 33]. Higher degrees of expansion (Cmax) and/or longer durations of expansion (area under the curve, AUC) of CAR-T cells in patients have been identified as biomarkers associated with positive clinical responses [34, 35]. On the other hand, CAR-T cells with insufficient potency may be ineffective, whereas an overly potent product can lead to toxicity, such as cytokine release syndrome (CRS) or on-target, off-tumor toxicity, including neurological (ICANS) and hematological (ICAHT) toxicities. Moreover, prolonged in vivo persistence of CAR-T cells has been correlated with the depth and durability of clinical responses [32, 36].

Currently, the most performed potency test is the quantification of interferon-gamma (IFN-γ) production after CAR-T cell stimulation. Recently, the percentage of CAR-positive T lymphocytes (transduction efficiency) and cell viability have also been classified as potency assays under certain regulatory frameworks, particularly by the FDA. Indeed, the FDA has recently drafted standardized expectations for CAR-T cell products. In particular, lot release criteria for early-phase Investigational New Drug (IND) applications do not require fully validated potency assays and specifications. However, such assays must be included when generating data in support of a Biologics License Application (BLA) (Center for Biologics Evaluation and Research, 2022a).

### Potency test methods for assessing the functional activity of CAR-T cells

#### Assessment of cytotoxic activity

After several hours of co-culture between CAR-T cells and target antigen-expressing cells, CAR-T cell cytotoxic activity can be assessed in two different ways. The first approach involves measuring target cell lysis. Currently, four techniques can be used for this measurement: Chromium-51 release, bioluminescence (luciferase-luciferin reaction), enzymatic release assays, impedance-based assays, and flow cytometry. Each method has its own advantages and limitations [37].

The second approach provides an indirect assessment of cytotoxic activity by detecting surface expression of CD107a/b on CAR-T cells via flow cytometry. This marker is upregulated following the degranulation of cytotoxic molecules such as perforins and granzymes.

After analyzing these methods, the working group concluded that target cell lysis measurement is the most relevant approach for evaluating CAR-T cell cytotoxic activity. Furthermore, to ensure broad implementation across French cell therapy units using existing equipment, flow cytometry was identified as the preferred technique. However, the feasibility of this choice depends on laboratory access to flow cytometers, which may not be universally available.

#### Assessment of proliferative capacity

Assessing the proliferative capacity of CAR-T cell preparations is a relevant parameter for CAR-T cell efficacy and toxicity in patients, as studies have shown that higher expansion levels (Cmax) and/or longer expansion durations (area under the curve, AUC) in patients are biomarkers associated with improved clinical responses [38].

After analyzing the advantages and limitations of available techniques for evaluating this biological function, the working group concluded that the most feasible approach would be a proliferation assay based on fluorochrome labeling (e.g., CFSE) and flow cytometry analysis. However, the group does not propose this test as a potency assay, as it requires a minimum of 3 to 4 days of CAR-T cell culture following antigen-specific stimulation. This extended timeframe is incompatible with the release of fresh CAR-T products.

#### Assessment of cytokine production

The production of one or more cytokines following co-culture of CAR-T cells with their specific antigen can be measured between 4- and 24-hours post-stimulation, depending on cytokine production kinetics. This assessment can be performed using three (or four) major assay types:

- Intracellular staining with flow cytometry,
- Cytokine quantification in co-culture supernatants via ELISA,
- ELISpot assay,
- Luminex technology, which combines ELISA with flow cytometry for multiplexed detection of multiple protein and/or nucleic acid targets.

Each of these techniques has advantages and limitations. Currently, commercially available CAR-T cell products with MA are released based on IFN-γ quantification. The working group emphasizes that this test is only relevant if it specifically quantifies CAR-dependent IFN-γ production. To ensure specificity, the assay should include a control mechanism, which can be based on:

- effector cells (using either CAR-negative patient T lymphocytes or, ideally, T cells expressing an irrelevant CAR),
- and/or target cells (using the same cell line with and without the CAR target antigen).

Ideally, both approaches should be included. Since obtaining additional patient-derived T lymphocytes is incompatible with clinical-scale CAR-T cell production, the working group recommends using target cell lines that are both positive and negative for the CAR antigen. This approach allows for the detection of CAR-specific IFN-γ production while distinguishing specific CAR activity from alloreactivity, which occurs when T cell receptors (TCRs) recognize major and minor histocompatibility complex differences between cell lines.

Thus, the proposed test should include four conditions.

1. No stimulation (negative control).
2. Stimulation with a cell line expressing the CAR target antigen.
3. Stimulation with a cell line lacking the CAR target antigen.
4. Non-specific polyclonal stimulation (e.g., anti-CD3 antibodies, anti-CD3/CD28 beads, PHA-L, etc.).

### Group recommendations

The objective of this workshop is to identify a potency assay that can be implemented across all academic units while meeting the following criteria: simplicity, compatibility with automated equipment, and use of a technique already used in hospital immunology laboratories. Additionally, since most batches will be released fresh, the assay must be rapid. Therefore, in addition to cell counting/viability assessment and determination of the percentage of transduced cells, the group has chosen to develop and validate a potency assay based on IFN-γ quantification using ELISA or ELISpot. This choice is driven by the relative ease of implementation compared to cytotoxicity or proliferation assays, as well as the fact that all currently approved commercial CAR-T products are released based on IFN-γ quantification.

IFN-γ measurement is performed following antigen-specific stimulation of the CAR-T cell preparation. Several types of stimulatory agents can be used, including:

- recombinant protein [39],
- beads coated with the CAR target antigen,
- human or murine [40] cell lines expressing the CAR target antigen.

The advantages and limitations of each of these stimulatory agents are summarized in Tables 4 and 5.

**Table 4:** Comparison of the advantages and limitations of different stimulatory agents for CAR-T cell preparations.

Stimulatory Agent	Signal	Advantages	Limitations	Remarks
**Recombinant Protein**	Restricted antigen	Simple, quantification of antigen number possible	Low stability (3 months) and cost (€350 for 50 µg, approx. €50/test)	Use of an irrelevant protein as a control
**Coated Beads**	Restricted antigen	Simple, quantification of antigen number possible	Low stability (3 months after reconstitution and storage at −80 °C) and cost (€1 300 for 2 mg, approx. €7–8 per well, €70–80/test)	Use of irrelevant beads as a control
**Allogeneic Human Cell Line**	Antigen-dependent and independent	Immunological synapse, mimics target in indication	Alloreactivity	Need to subtract alloreactivity with antigen-negative cell line
**Murine Cells**	Antigen-dependent and independent	Immunological synapse	Potential xenogeneic reactivity (not detected under test conditions)	Use of antigen-negative cell line as control

**Table 5:** Comparison of ELISA and ELISpot techniques for IFN-γ quantification.

	ELISpot	ELISA
**Possible quantification of IFN-γ producing CAR-T cells**	Yes	No
**Quantifiable data provided**	Number of spots for 10 000 or 100 000 cells	Concentration in pg/mL
**Number of CAR-T cells required per condition**	Approximately 10 000 to 100 000	Approximately 10 000 to 100 000
**Possible stimulation methods**	Recombinant protein/beads/cell lines	Recombinant protein/beads/cell lines
**Effector:Target ratio commonly used**	At least 3 ratios (0.1/1, 1/1, and 1/10)	At least 3 ratios (0.1/1, 1/1, and 1/10)
**Incubation time**	Most often 4 to 24 h	Most often 16 to 24 h
**Cost**	- Around €200 to €400 for a pre-coated 96-well plate - Approximately €50/test when using strips, but well coating needs to be validated internally with common references across labs	- Around €200 to €800 for a pre-coated 96-well plate - Approximately €60/test when using strips, but well coating needs to be validated internally with common references across labs

Following the analysis of the advantages and limitations of stimulatory agents and the two techniques, the working group will likely recommend the use of coated beads with the antigen or murine cells modified to express the target antigen, which were developed by Jean-Baptiste Latouche’s team [40], based on the results of a preliminary comparative study.

While the ELISpot test provides additional quantitative data (number of cells capable of producing IFN-γ), it is more complex and costlier (not universally available in all labs) compared to the ELISA technique. Therefore, the working group will likely propose conducting an ELISA test with an 18- to 24-hour incubation period to align with the end time of the manufacturing process and the working hours of technical staff.

A first comparative study (ELISA versus ELISpot, with different stimulatory agents) will be conducted by the biotherapy laboratories. Based on what has been done and the results of this study, recommendations will be issued for all biotherapy laboratories in future workshop.

## Conclusion

This position paper aims to harmonize QC procedures for academic autologous CAR-T cell production. The goal is to standardize QC testing for batch release in academic cell therapy units, enabling faster, cost-effective production and the use of fresh CAR-T cells. Key QC measures include mycoplasma detection, endotoxin testing, vector copy number quantification, and potency assessment. This work emphasizes method validation and standardized testing to ensure the safety and efficacy of academic CAR-T cell therapies.

## Unanswered questions and further research to do in the field

- Sterility testing and characterization/identity/purity assessments will be the topic of another workshop.
- The group also wishes to propose a technique for assessing cytotoxic activity using flow cytometry through the measurement of the lysis of allogeneic or xenogeneic target cells, as the use of autologous tumor cells in a standardized way is not feasible (potency assay per se).
- The evaluation of the proliferative capacity of CAR-T cells by flow cytometry will be the third control to be implemented to fully characterize the functioning of these cells.
- Evaluation of the importance QC harmonization for manufacturing and patient immune monitoring using advanced flow cytometry, omics, RNAseq [41].
