# Correction: Harmonisation of quality control tests for academic production of CAR-T cells: a position paper from the WP-bioproduction of the UNITC consortium

**DOI:** 10.1038/s41409-025-02672-5

**Published:** 2025-06-30

**Authors:** Chrystel Marton, Béatrice Clémenceau, Guillaume Dachy, Clémence Demerle, Sophie Derenne, Christophe Ferrand, Camille Giverne, Jean-Baptiste Latouche, Ludovic Lemée, Jérémie Martinet, Halvard Bonig, Danièle Bensoussan, Christian Chabannon, Ulrike Köhl, Marina Deschamps, John De Vos, Jean-Sébastien Diana, Aurore Dougé, Edouard Forcade, Jeanne Galaine, Stéphanie Thiant, Anne Galy, Jérôme Larghero, Loïc Reppel, Sébastien Viel, Olivier Boyer, Ibrahim Yakoub-Agha

**Affiliations:** 1 CHU Lille, Univ. Lille, Inserm, U1286 - INFINITE - Institute for Translational Research in Inflammation, F-59000 Lille, France; 2 Unité de Thérapie Cellulaire et Génique (UTCG), CHU de Nantes, Nantes, France; 3 Hematology department, Cliniques Universitaires Saint-Luc, Brussels, Belgium; 4 Centre de Thérapie Cellulaire, Institut Paoli-Calmettes (IPC) & module Biothérapies du Centre d’Investigations Cliniques de Marseille, Inserm CBT-1409 / Aix-Marseille Université / AP-HM / IPC, Marseille, France; 5 Etablissement Français du Sang, La Plaine Saint Denis, Saint-Denis, France; 6 Université de Franche-Comté, EFS, INSERM, UMR RIGHT, F-25000 Besançon, France; 7 CHU de Rouen, Laboratoire d’Immunologie et de Biothérapies, Université de Rouen Normandie, Inserm, UMR 1234, F-76000 Rouen, France; 8 Univ Rouen Normandie, Inserm, UMR 1234, CHU de Rouen, Department of Immunology and Biotherapy, F-76000 Rouen, France; 9 CHU de Rouen, Department of Virology, F-76000 Rouen, France; 10 Goethe University, Faculty of Medicine, Institute for Transfusion Medicine and Immunohematology, Frankfurt a.M, Germany; 11 CHRU de Nancy, Unité de Thérapie cellulaire et Banque de Tissus (UTCT). Université de Lorraine, UMR 7365 IMoPA, Vandoeuvre-les-Nancy, France; 12 Fraunhofer Institute for Cell Therapy and Immunology as well as Institute for Clinical Immunology, University of Leipzig, Leipzig, Germany; 13 Department of Cell and Tissue Engineering, University Montpellier, CHU Montpellier, F-34000 Montpellier, France; 14 APHP, Hôpital Universitaire Necker Enfants Malades, Centre d’Investigation Clinique en Biothérapie, Laboratoire de Thérapie Cellulaire et Génique, Paris, France; 15 CHU de Clermont-Ferrand, Service Oncologie médicale, EA 7453 Chelter Université Clermont Auvergne, 63000 Clermont-Ferrand, France; 16 CHU de Bordeaux, Service Hématologie Clinique et Thérapie Cellulaire, UMR 5164, F-33000 Bordeaux, France; 17 Division of hematology, Oncology and transplantation, department of medicine, Maisonneuve-Rosemont hospital, Montréal, QC Canada; 18 ART-TG, Inserm, US35, 91100 Corbeil-Essonnes, France; 19 AP-HP, Université Paris Cité, Hôpital Saint-Louis, Centre MEARY de Thérapie Cellulaire et Génique, Unité de Thérapie Cellulaire, Centre d’Investigation Clinique en Biothérapies, Inserm, Paris, France; 20 Plateforme de biothérapies - Médicaments de Thérapie Innovante, Hôpital Edouard Herriot, Hospices Civils de Lyon, 69003 Lyon, France

**Keywords:** Gene therapy, Cancer therapy

Correction to: *Bone Marrow Transplantation* 10.1038/s41409-025-02637-8, published online 29 May 2025

The author name Marina Deschamps has been initially written as Marine Deschamps.

The original article has been corrected.

